# Complexities related to the amorphous content of lactose carriers

**DOI:** 10.1016/j.ijpx.2023.100216

**Published:** 2023-10-23

**Authors:** Pauline H.M. Janssen, Lorina M.N. Bisharat, Marly Bastiaansen

**Affiliations:** a Department of Pharmaceutical Technology and Bio pharmacy, University of Groningen, Antonius Deusinglaan 1, Groningen 9713 AV, the Netherlands; b DFE Pharma GmbH & Co. KG, Klever Str. 187, Goch 47574, Germany; c Department of Pharmaceutics and Pharmaceutical Technology, School of Pharmacy, The University of Jordan, Amman 11942, Jordan

**Keywords:** Amorphous content, Control strategy, Dry powder inhalation, Interaction forces, Lactose, Physical characterization, Polymorphic form

## Abstract

Although the amount of amorphous content in lactose is low, its impact on the performance of a dry powder inhalation formulation might be high. Many formulators and regulatory agencies believe that the levels of amorphous content should be controlled once there is a relationship with the final product performance. This is however not an easy task. The current paper elaborates on multiple challenges and complexities that are related to the control of the amorphous content in lactose. The definition and quantification methods of amorphous lactose are reviewed, as well as challenges related to thermodynamic instability. Additionally, current monographs and recent position papers considering this parameter are discussed to provide an overview of the regulatory landscape. Development of a control strategy is recommended, provided that the amorphous content at a specific moment in the process has shown to have an impact on the performance of the dry powder inhaler.

## Highlights

• The attention of regulatory agencies and formulators to amorphous content has increased.
• The apparent amorphous content in lactose is difficult to define.
• Different quantification methods can result in different values.
• The apparent amorphous content can be unstable over time.
• Control strategies should only be developed when the amorphous content has an effect on the final product performance.

## Graphical abstract

**Figure:**
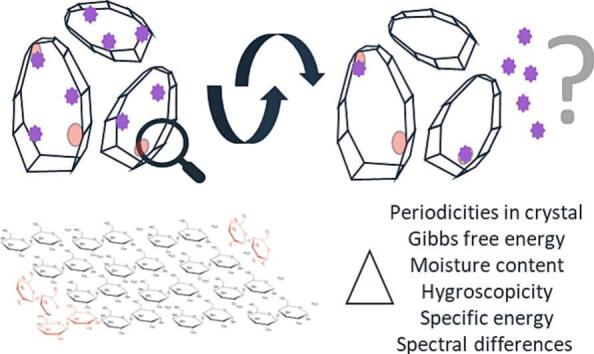

## 1 Introduction

Apparent amorphous lactose is a collective term used for all phases of lactose that do not have a defined crystalline structure. Over the last years, the apparent amorphous content of inhalation grade lactose gained interest from the pharmaceutical industry (FDA, 2018; Moreton et al., 2020). Many formulators and regulatory agencies believe that the levels of amorphous content should be controlled, once there is a proven relationship with the final product performance. This is however not an easy task, due to the unstable nature of amorphous and the complexity of quantification techniques.

Many different methods exist to evaluate the apparent amorphous content in a lactose sample. All these methods however, face the challenge that amorphous refers to a broad set of types of disorder in crystal structures with variable material properties (Lehto et al., 2006).

It can be difficult for formulators to determine the apparent amorphous content range that leads to acceptable product quality. This is, amongst others, related to the unstable character of amorphous lactose. The amorphous content in lactose is thermodynamically unstable due to the high energy associated (Huppertz and Gazi, 2016; Wijayasinghe et al., 2020). Dependent on the temperature and humidity, amorphous lactose is susceptible to crystallization. Besides the unstable character of amorphous lactose, performing structured analyses is challenging as samples that only vary in amorphous content are hard to get. Apparent amorphous content is not an independent parameter, and thus steering on this parameter in isolation is hardly possible.

The current monographs for lactose described in the European Pharmacopeia (EP), United States Pharmacopeia- National Formulary (USP-NF), Japanese Pharmacopeia (JP), Indian Pharmacopeia (IP) and the Chinese Pharmacopeia (CP) do not describe any mandatory testing for the apparent amorphous content (*Lactose Hydrate Monograph*, 2022; Lactose Monohydrate Monograph, 2021; Lactose Monograph, 2022; Lactose Monohydrate Monograph, 2020; Lactose Monohydrate Monograph, 2023). The parameter is however in scope of many discussion on future regulatory frameworks, including position papers and the US FDA draft guidance for industry (Moreton et al., 2020; Lactose for inhalation for CP2025, 2022).

All in all, there are many complexities associated with the apparent amorphous content in lactose. It is a challenging parameter that has the attention of the industry, and an in-depth understanding and clear agreements between drug authorization holders and suppliers is critical.

This article elaborates on multiple complexities associated with the apparent amorphous content in inhalation grade lactose. Section 2 introduces carrier-based dry powder inhalation (DPI) and the importance of interaction forces between an active pharmaceutical ingredient (API) and a carrier. Different polymorphic forms of lactose, including the amorphous form and its thermodynamically unstable character, are introduced. Section 3 evaluates the influence of amorphous content on the performance of lactose carriers. In section 4 the complexities and differences between different methods used to quantify the apparent amorphous content are reviewed. Section 5 describes Quality by Design and control strategies to mitigate the risk of variation in performance of dry powder inhalation as a result of variation in the apparent amorphous content. Chapter 6 reviews the regulatory landscape related to the amorphous content in inhalation grade lactose. The section highlights the current pharmacopeial standards and proposed changes on amorphous content in lactose for inhalation. Section 7 concludes the previous sections.

## 2 Carrier-based dry powder inhalation

Dry powder inhalers (DPIs) are compact and portable devices that are designed to deliver medication in the form of a dry powder to the lungs. Pulmonary delivery is non-invasive and patient-friendly. It provides a rapid and predictable onset of action, due to the large surface area of the lungs that is available for absorption (Anderson et al., 2022). Additionally, lower doses can be required to reach the local target effective dose, due to the avoidance of the first-pass effect of the gastrointestinal tract and the liver (Patton et al., 2004; Chen et al., 2020).

### 2.1 Formulation

The efficacy of DPIs relies on the deposition of the API into the lungs, which is predicted in-vitro by the aerodynamic particle size distribution (APSD). The APSD defines how powders behave in an airstream during inhalation. It depends amongst others on the size, shape and density of particles. The optimal aerodynamic particle size to reach the bronchial region is below 5 μm, with the majority of the particles being 2–3 μm (Peng et al., 2016). Crystalline API particles are therefore generally too large and require size reduction, like can be achieved with micronization. Micronized particles however, have a high surface area and high surface energy. Hence high cohesive and adhesive forces are present, resulting in flowability and dispersibility challenges. The most common approach to address these challenges is to formulate the micronized API with a carrier excipient. The carrier has coarser particles than the API, typically between 50 and 100 μm in diameter, and forms a loose bond with the drug (Peng et al., 2016).

Drug delivery is initiated by a quick single inhalation event by a patient through the device, which actuates the dispersion of the formulation into the inhaled air. The energy of the inspired air flow drives the detachment of the drug particles from the surface of the carrier, so drug particles can be deposited into the lungs. Successful drug delivery by carrier-based DPIs depends on many factors. These factors include amongst others the device, the filling platform, the drug or active pharmaceutical ingredient (API), the carrier and the blending process (De Boer et al., 2012). The balance between adhesive and cohesive forces should be adjusted to a level that enough adhesion is present to manufacture a stable formulation, yet easy separation is allowed during inhalation (Pilcer and Amighi, 2010). The interaction forces between drug and carrier are typically dominated by physical forces. These forces include van der Waals forces, electrostatics, capillary forces, and interlocking (Telko and Hickey, 2005). The magnitude of these forces varies with many factors like particle size, shape, surface properties, and contamination of the carrier particle and relative humidity (Hickey et al., 2007).

### 2.2 Lactose as a carrier

Lactose is the most widely accepted carrier for DPI formulations in the market (Hebbink et al., 2022; Rahimpour and Hamishehkar, 2012). The advantages of using lactose as a carrier are numerous. Lactose is a natural, soluble excipient with good physical and chemical stability (Villalobos-Hernández and Villafuerte-Robles, 2001). It has a long historic track-record resulting in a large set of (favorable) toxicology and safety data (Baldrick and Bamford, 1997). Additionally, it is well accepted by authorities and it is available with well controlled particle size and flow properties. This versatility of lactose can be exploited to optimize each formulation towards the desired performance (Hebbink et al., 2022; Pilcer et al., 2012). Extrinsic addition of lactose fines to a coarse lactose carrier for example, significantly enhances the aerosolization performance and delivery of the API (Kinnunen et al., 2014).

Marketed inhalation lactose grades typically consist mostly of α-lactose monohydrate, which is the most stable polymorph of lactose. Other crystal structures, like β-anhydrous lactose, stable anhydrous α-lactose and hygroscopic anhydrous α-lactose however also exist (Kirk et al., 2007). The different polymorphs differ in properties like stability, hygroscopicity, crystal structure, shape, surface energetics, and chemical reactivity (Kirk et al., 2007; Della Bella et al., 2016). Amorphous lactose is a collective term that is used for all phases of lactose that do not have a defined crystalline structure. It is therefore a thermodynamic unstable phase that encompasses many different types of structural disorder, each with its own properties (Price and Young, 2004). Structural disorder could for example vary in the frequency and scale of crystal disruptions (Peltonen and Strachan, 2020). Additionally, local disorder could be present as a result of anisotropic lattice distortions, amorphous surface layers, mutarotation, particle size reduction disruptions and crystal defects (e.g. vacancy, vacancy dislocation, substitutional impurity, interstitial dislocation loop, dislocation, interstitial self-atom, interstitial impurity) (Garnier et al., 2008; Föll, 2019).

**Fig. 1:**
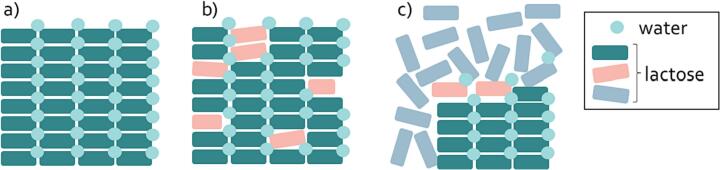
Graphical representation of an intact lactose monohydrate crystal (a), a lactose monohydrate crystal with type II apparent amorphous regions (red rectangles) formed upon milling (b), and a lactose monohydrate crystal with type I amorphous lactose (blue rectangles) formed upon drying (c). (For interpretation of the references to colour in this figure legend, the reader is referred to the web version of this article.)

Even though marketed inhalation grades are classified as α-lactose monohydrate, small fractions of amorphous lactose in these materials cannot be excluded. The crystalline state of lactose monohydrate can be inadvertently rendered amorphous by the introduction of varying degrees of stress (Newman and Zografi, 2014). Stresses that can generate layers of amorphous lactose include stresses from drying and comminution operations (Bronlund and Paterson, 2004; Savolainen et al., 2010). Amorphous surface layers that originate from drying processes are categorized as type I apparent amorphous lactose. Type I amorphous lactose layers typically form during the crystallization process of lactose. Adhering solvents after filtration may cause dissolved lactose to remain at the surface of the crystals. During drying, these lactose molecules do not get the time to crystallize and therefore will appear in a completely random structure, that is referred to as an apparent amorphous domain (Dominici et al., 2022). Down-stream sieving, milling or micronization processes of lactose monohydrate could also result in apparent amorphous domains (typically denoted as type II apparent amorphous). In this case, the energy that is applied to reduce the size of lactose particles disrupts the crystal phases of the molecules (Pazesh et al., 2017; Badal Tejedor et al., 2018). Fig. 1 shows a graphical representation of these two types of amorphous lactose in a lactose monohydrate crystal. It is worth highlighting that the different types of amorphous lactose are not always very distinct – and also intermediates or different types of disorder can exist.

### 2.3 The unstable character of amorphous lactose

**Fig. 2:**
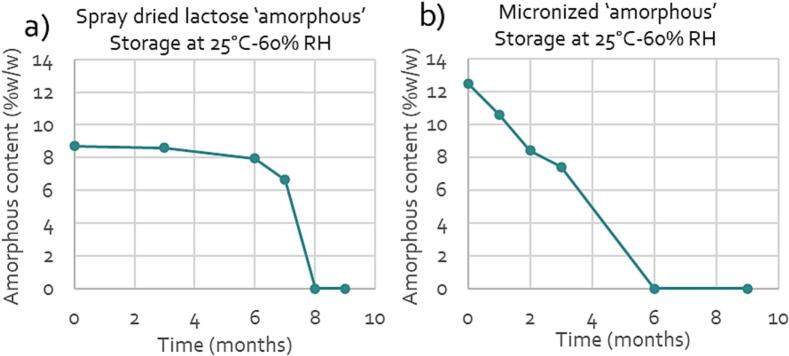
The amorphous content over time for a) a spray dried grade of lactose (containing mainly type I apparent amorphous) and b) micronized lactose (containing mainly type II apparent amorphous from comminution) (DFE Pharma GmbH and Co. KG, 2023).

Amorphous lactose is thermodynamically unstable in nature due to the high energy associated with this form. There is a thermodynamic drive to reduce the energy of the system typically leading to recrystallization (Huppertz and Gazi, 2016). This recrystallization is highly impacted by internal and external conditions. These factors include the type of amorphous structure (Haque and Roos, 2005), the presence of impurities (Wijayasinghe et al., 2020), the temperature and the relative humidity (Clark et al., 2016). Packaging, transport and intermediate opening of packaging could play a crucial role in the amount of crystallization. The predictability of the speed and amount of crystallization is therefore typically very low. Fig. 2 shows an example of recrystallization of two lactose grades with different initial amounts and types of apparent amorphous lactose.

Both graphs show a decrease in the apparent amorphous content over time, due to recrystallization. Based on this observation, it becomes clear that the amorphous content is a material property that is variable over time. Therefore, drawing relationships between the amorphous content and the performance of lactose can only be done based on actual data on the apparent amorphous content available. This can only be achieved when the determination of amorphous content is performed at the time that the material is used in the formulation. Data on the apparent amorphous content directly after production of a raw material is not representative as it does not reflect the amorphous content upon use.

## 3 Influence of amorphous content on the performance of lactose carriers

Although the amount of apparent amorphous content in lactose is low, its potential impact is high. This is related to the typical location of apparent amorphous lactose, which is at the surface of the particles (Newell et al., 2001a). Especially in inhalation applications, the presence of amorphous lactose at the surface is thought to be a factor that can have a potential impact on the interaction forces with the API (Buckton and Darcy, 1995). Other factors that can impact the interaction forces include the particle size distribution, density, porosity, surface morphology, surface energy, and impurity level (Peng et al., 2016; Telko and Hickey, 2005; Shetty et al., 2020; French et al., 1996; Zhang et al., 2019).

### 3.1 Chemical stability

Amorphous lactose is the most reactive solid state form of lactose (Gänzle et al., 2008). Amorphous lactose has reduced chemical stability, explained by the increased surface area, increased hygroscopicity and molecular mobility. Amorphous lactose can contain hundred times more free moisture than the crystalline form (Bronlund and Paterson, 2004). It is hygroscopic and when sufficient water is absorbed, molecular mobility is enhanced (Huppertz and Gazi, 2016). Molecular mobility is thought to be one of the most important factors determining the lower chemical stability of amorphous materials (Yoshioka and Aso, 2007). Additionally, the molecular arrangement is less dense in amorphous materials, with steric hindrance from the three-dimensional molecular structure in crystalline solids (Vollenbroek et al., 2010).

Ranger et al. (2017) investigated the non-enzymatic browning kinetics of amorphous and crystalline lactose. They showed that amorphous lactose has more intensive browning under similar conditions compared to crystalline lactose, due to the increased surface area available (Ranger et al., 2017). Similarly, Qiu et al. (2005) observed higher Maillard reaction rates when pure amorphous lactose was used compared to spray dried lactose monohydrate or anhydrous lactose (Qiu et al., 2005).

### 3.2 Physical stability

In the crystalline state, molecules are organized in a defined tri-dimensional order which limits their motion. An amorphous structure in contrast, exhibits molecular disorder which can be sensitive to physicochemical changes when exposed to stress. Therefore, crystalline materials have higher physical stability compared to amorphous materials (Palzer, 2009). Due to the hygroscopic character of amorphous lactose, it could easily absorb moisture. This increase in moisture content can result in increased cohesiveness and increased caking ability of a powder (Huppertz and Gazi, 2016; Stankovic-Brandl et al., 2022; Das et al., 2010). Additionally, amorphous regions can recrystallize over time under the influence of water. Consequent physical changes can be observed, including sticking, caking and lumping (Intipunya and Bhandari, 2010). Stickiness and caking is typically controlled by handling and storing under dry conditions (Listiohadi et al., 2009).

Recrystallization of amorphous regions under the influence of water also have shown to affect the interaction between carrier and drug (Lu et al., 2017; Young et al., 2007; Young et al., 2003; Depasquale et al., 2015). Even very small amorphous fractions on the surface of crystalline lactose can influence its surface energy and consequent interparticle interactions of the API (Buckton and Darcy, 1996). Agglomeration of drug particles by recrystallization directly affects the aerosolization performance. For example, a strong reduction was observed in the efficiency of the Symbicort Turbuhaler and Seretide Diskus formulations when devices were stored at 75 %RH at 40 °C compared to an inhaler stored at 25 °C and 30% RH (Borgström et al., 2005).

### 3.3 Conditioning

Crystallization of amorphous lactose can be suppressed by reducing temperature and humidity below the glass transition temperature of the material. However, it has recently been shown that, although molecular mobility in amorphous material is greatly reduced below the transition point, it is not completely halted (Lourdin et al., 2002).

To overcome long-term stability issues due to recrystallization, powders can be conditioned at controlled RH and temperature to allow the conversion of amorphous to crystalline material prior to blending of the drug and carrier (Brodka-Pfeiffer et al., 2003). A recent study showed that conditioning at high RH reduced the amorphous content in the lactose. However, agglomeration of fine particles reduced the dispersibility of the powder (Stankovic-Brandl et al., 2022). An extensive review of the physical stability of DPI formulations was recently published by Shetty et al. (Shetty et al., 2020).

When apparent amorphous content crystallizes before it is blended into a formulation, limited impact on the formulation by the historical presence of this fraction is expected. De Boer et al. evaluated the impact of amorphous lactose in the carrier residue in a classifier-based test inhaler with 4% budesonide (De Boer et al., 2012). No significant difference on the carrier residue was observed for formulations containing lactose monohydrate fines or recrystallized amorphous fines. Using amorphous fines however resulted in significantly higher carrier residues via changes in the capillary forces and the formation of solid bridges. This was only observed when the amorphous fines recrystallized after they were added to the blend.

## 4 Analytical techniques to quantify amorphous content

Different analytical techniques exist to evaluate the apparent amorphous content of lactose monohydrate. These techniques are based on differences in several material properties between crystalline and amorphous lactose. Some of the most commonly used techniques for quantification are discussed below.

### 4.1 Powder X-Ray diffraction

Powder X-Ray Diffraction (PXRD) is typically used to identify and quantify the different crystalline phases in lactose. This method is based on measuring periodicities of molecules in a powder sample that result in specific diffraction patterns. The shape of diffractions peaks is affected by a variety of parameters, including the size of crystallites making up the particles (Chen et al., 2001). Sharp peaks indicate fully crystalline structures, and diffraction peaks broaden when the crystallite size is reduced (Shah et al., 2006). Due to the completely random structure in amorphous lactose, broad peaks (often referred to as halos) are visible in the PXRD. Deconvolution of the PXRD pattern into sharp peaks and broad peaks allows for the determination of amorphous content (Montoya-Escobar et al., 2022). Main challenges of quantification of amorphous content with this technique relate to the peak deconvolution and the definition of the cut-off peak broadness for amorphous content.

### 4.2 Isothermal microcalorimetry and solution calorimetry

The quantification of apparent amorphous content with isothermal microcalorimetry (IMC) is based on the difference in Gibbs free energy (ΔG) of amorphous and crystalline states of lactose (Katainen et al., 2005). During a measurement of a sample, the humidity level of the sample is increased to plasticize the amorphous fractions. By a reduction of the activation energy, spontaneous crystallization is evoked. The exothermic energy resulting from crystallization is measured and is proportional to the apparent amorphous content of the material (Angberg, 1995). Quantification in this case is performed by comparing to a reference standard of 100% amorphous lactose.

Solution calorimetry (SC) is also based upon the difference in Gibbs free energy (ΔG) between amorphous and crystalline states of lactose (Hogan and Buckton, 2000). The Gibbs free energy in this case is measured relative to the energy state in a solution. Amorphicity determinations are based upon the principle that the heat of dissolution is proportional to the amorphous content (Hogan and Buckton, 2000).

### 4.3 Thermal analysis by differential scanning calorimetry

Differential Scanning Calorimetry (DSC) can be used in multiple ways to quantify the amorphous content. Conventional DSC experiments have a linear heating ramp. In this case the sample is heated above the glass transition temperature to evoke crystallization (Gombás et al., 2002). The exothermic energy resulting from crystallization is measured and is proportional to the amount of apparent amorphous content of the material.

A requirement for the crystallization towards lactose monohydrate is that sufficient water molecules are present to be incorporated in the crystal structure. Water molecules are typically already present in amorphous lactose in the form of free water. Additionally, a moisturizing agent (e.g. acid casein) can be added to the sample, which simultaneously has a plasticizing effect on the lactose. If limited moisture is present, it is likely that partial crystallization towards anhydrous lactose takes place – which might correspond to a different polymorphic form with a different energy state.

DSC techniques can also be used to measure the glass transition (Tg) response. High speed DSC or hyper DSC (HDSC) is in particular a suitable technique to measure the Tg response and can therefore be used to quantify the amorphous content (Saunders et al., 2004; Gabbott et al., 2003). The step height change in heat flow at the Tg is linearly related to the amorphous content. The glass transition response is seen as a change in heat capacity and depends on the amount of apparent amorphous content in the sample (Lappalainen and Karppinen, 2010).

One of the challenges associated with calorimetric methods like DSC and ISM methods is related to the different levels of disorder, and therefore the different energy states that amorphous lactose can have. Measurement results indicate the total amount of exothermic crystallization energy, but the crystallization energy depends on the amount of structural disorder. As it is impossible to differentiate between the different types of amorphous, it is impossible to select a reference measurement that indicates 100% amorphous (Ramos et al., 2005). The calibration curve of the matching type of amorphous content is therefore based upon a chosen reference type of amorphous.

### 4.4 Dynamic Vapor Sorption (DVS)

Dynamic Vapor Sorption is a gravimetric sorption technique that measures the absorption of a solvent (typically water) by a sample. Samples are exposed to different relative humidity (RH) values to quantify the hygroscopicity of the material – which is typically different for amorphous compared to crystalline lactose (Bronlund and Paterson, 2004). Additionally, exposure to humidity can plasticize the amorphous lactose and trigger crystallization towards lactose monohydrate. The weight gain after the crystallization indicates the amount of moisture molecules that are incorporated in a lactose monohydrate crystal and is therefore a measure of the initial apparent amorphous content (Vollenbroek et al., 2010). With DVS measurements, different types of amorphous content can be distinguished by the relative humidity level at which they crystallize.

One of the major challenges of DVS is that it based on the assumption that all crystallization events indicate crystallization from amorphous lactose to lactose monohydrate. Other structural changes, like crystallization of anhydrous lactose to lactose monohydrate might interfere with the measurements.

### 4.5 Thermal gravimetric analysis

Thermal Gravimetric Analysis (TGA) can also be used to estimate the crystallinity of a lactose sample. Peaks and mass changes on a thermogram are characteristic of surface water, water of crystallization and amorphous lactose (Smith et al., 2015). When TGA is used to quantify the proportion of crystalline material, one has to assume that the water of hydration represents the amount of crystalline lactose. However, hydration water of partially amorphous particles will behave differently, and can result in uncertainty in the estimated of crystallinity so derived (Chidavaenzi et al., 1997).

### 4.6 Dynamic mechanical analysis

Dynamic Mechanical Analysis (DMA) can be used to evaluate the mechanical properties of a sample as function of the temperature (Duncan and Price, 2023; Royall et al., 2005). Oscillating stress is applied to a sample, resulting in deformation within the sample. The mechanical properties of amorphous materials are substantially different than for crystalline materials. The deformation is therefore a measure of amorphicity of the sample.

### 4.7 Inverse gas chromatography

Inverse Gas Chromatography (IGC) is a technique that is well suited to study the amorphous surface of powders (Newell et al., 2001b). In IGC, a vapor of probe molecule is injected into a column packed with the solid sample under investigation at a fixed gas flow rate. The retention time of the probe molecule is a measure for in the specific energy of the sample (Newell and Buckton, 2004). Important to note is that IGC measures the polymorphic composition on the surface of the samples. In samples with 20% *w*/w amorphous content for example, Buckton et al. did not detect the crystalline form at all, giving data as if the entire surface was amorphous (Buckton and Gill, 2007).

### 4.8 Spectral techniques

Raman, Near Infrared Spectroscopy (NIRS), and solid-state Nuclear Magnetic Resonance (ss-NMR) techniques are based upon spectral differences (Murphy et al., 2005; Buckton et al., 1998; Gustafsson et al., 1998). NIRS is based on light absorption of the sample. Crystalline and amorphous lactose have slightly different absorption energies related to the stretching and vibration of different molecular bonds (Gombás et al., 2003).

### 4.9 Comparison of methods

Besides the explained methods, many other methods for the quantification of the apparent amorphous content in lactose exists. Each of these methods is based upon a material property that is substantially different for crystalline lactose than for amorphous lactose. Table 1 provides an overview of the different techniques, the measured material property. An overview of different methods and their advantages and disadvantages is provided by multiple authors (Lehto et al., 2006; Listiohadi et al., 2009; Shah et al., 2006; Lappalainen and Karppinen, 2010; Fix and Steffens, 2004).

**Table 1:** Overview of different techniques, the measured material property and the according detection limits.

Technique	Measured material property	Detection limit	Reference
X-ray Powder Diffraction (XRPD)	Periodicities in molecule structure	0.5%	(Chen et al., 2001; Fix and Steffens, 2004)
Isothermal Microcalorimetry (IMC)	Solution energy	0.3	(Buckton et al., 1995; Figura, 1993; Murti Vemuri et al., 2004)
Solution Calorimetry (SC)	Enthalpy of solution	1%	(Hogan and Buckton, 2000; Harjunen et al., 2004)
Differential Scanning Calorimetry (DSC)	Crystallization energy	1%	(Lappalainen and Karppinen, 2010)
High-speed DSC	Crystallization energy	<1%	(Saunders et al., 2004)
Dynamic Vapor Sorption (DVS)	Crystallization moisture	0.7%	(Hogan and Buckton, 2001)
Thermal Gravimetric Analysis (TGA)	Moisture content	1%	(Listiohadi et al., 2009)
Dynamic Mechanical Analysis (DMA)	Mechanical properties	2.8%	(Shah et al., 2006)
Near Infrared Spectroscopy (NIRS)	Absorption energy spectrum	0.5%	(Fix and Steffens, 2004)
Solid state nuclear magnetic resonance (^13^C NMR)	Nuclear spin of carbon atoms	<0.5%	(Gustafsson et al., 1998)
Inverse gas chromatography (IGC)	Specific surface energy	<1%	(Newell and Buckton, 2004; Ambarkhane et al., 2005)

A challenge related to all measurement methods is that there is typically not one distinct value for amorphous lactose, due to the many different types of disorder that can be referred to as the amorphous content. Different analytical techniques are inherently sensitive to different types of amorphous content or different length-range of disorder. Different techniques can therefore show different amorphous values for the same sample (Lehto et al., 2006; Peltonen and Strachan, 2020). Mah et al. for example evaluated the amorphicity of glibenclamide after various milling times with three different techniques (Mah et al., 2014). According to XRPD, the sample was completely amorphous after 30 min of milling. In contrast, the sample was completely amorphous after 60 min and 180 min of milling according to the Raman spectroscopy and DSC analyses, respectively.

Due to the inherent differences of different methods, apparent amorphous content values only provide information when discussed in relation to the used measurement method. This highlights that discussions on amorphous content can only be done with full understanding and clear agreements on the definition and measurement method for the apparent amorphous content.

## 5 Quality by design

One challenge that formulators might experience in the context of apparent amorphous content is the lack of a clear route for the confirmation if apparent amorphous content is impacting a specific formulation. Initial formulation and process development is typically focused on finding a combination of raw materials and process parameters that meets the target product profile. Once this combination has been found, projects typically progress to the formulation and process optimization stage. In this stage, it is evaluated which properties are critical for the product performance, which can eventually lead to raw material specifications. Specifications should include those properties and ranges necessary for the excipient to meet the final product quality requirements (IPEC, 2008).

Quality by Design (QbD) is a pro-active quality approach that is focused on the prediction of product performance based upon design inputs. It emphasizes that robust formulations and processes should be able to accommodate the typical variation seen in APIs, processes and excipients without compromising on the manufacture, stability or performance of the product. If QbD is properly executed, it leads to understanding of the range of input variables within which the process can operate without compromising on product quality. This range may be determined through Design of Experiment (DoE) techniques, focusing on the evaluation of independent parameters. During DoE evaluations, raw material properties are typically varied intentionally to understand their impact on product performance. Pharmaceutical manufacturers prefer to use samples with properties that represent the extremes in the intended specification range. While desirable, it is not possible for excipient manufacturers to provide such samples.

Ideally, the impact of apparent amorphous content should be tested with QbD samples containing high and low apparent amorphous content. The apparent amorphous content in a sample is however the result of many factors. Suppliers are therefore unable to actively steer or control this parameter to a high value. The apparent amorphous content in a sample is amongst others the result of the formation of apparent amorphous content during crystallization and comminution processes, and the removal of apparent amorphous content as a result of the environmental conditions and the way of handling and storage. Additionally, the apparent amorphous content from comminution processes is highly related to the final particle size – which is a key performance driver in DPI formulations. Low amorphous samples could be provided by exposing lactose to high relative humidity – but this could also affect other material properties, like particle size or surface smoothness. In conclusion, suppliers are unable to create QbD samples with the apparent amorphous content as an independent variable. Pharmaceutical manufacturers could however consider alternative methods to test the impact, including spiking with amorphous lactose, the use of samples from other similar pharmaceutical grades or extrapolation of data from smaller ranges.

Even though suppliers are unable to create QbD samples with predetermined amorphous content on purpose, samples with variable apparent amorphous content might be observed. All production processes, including the production processes for inhalation grade lactose monohydrate, have some inevitable degree of variation (Gorsky, 2019). Production processes can shift amongst others due to variation in conditions, human intervention, variability of raw material, equipment getting older, or variability of analytical instrumentation. This natural variability could result in samples with a slightly higher or lower amorphous content – that coincidently might be used for testing the impact of apparent amorphous content on performance. Main challenge for these samples remains the unstable nature of amorphous lactose, that results in variable and unpredictable amorphous content over time and uncertainty in the apparent amorphous content at the moment of use. Additionally, manufacturing lead times from powder till final dosage form can take weeks or months. The apparent amorphous content of the lactose could change during this manufacturing period. Thorough understanding of potential correlations can therefore only be obtained when the amorphous content is monitored throughout the entire process.

**Fig. 3:**
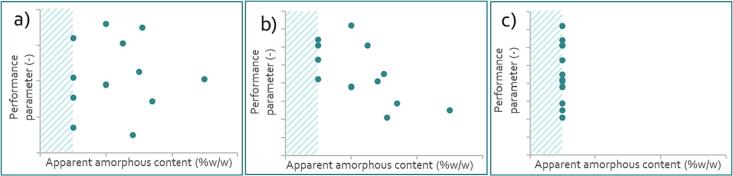
Theoretical correlation graphs between the apparent amorphous content and a performance parameter. The limit of detection for the amorphous content method was taken as value when no amorphous content was detected, as indicated by the striped area. a) shows a situation where apparent amorphous content is not a (key) driver for performance and no control strategy is required; b) shows that apparent amorphous content is a key driver for performance and therefore needs a control strategy; and c) shows formulations with an apparent amorphous content below the detection limit, providing no evidence for the need for a control strategy.

In the case pharmaceutical manufacturers have tested batches with variable apparent amorphous content at relevant timepoints during manufacture, conclusions can be drawn on the impact of apparent amorphous content on the performance. Three different trends could be observed, as indicated in Fig. 3, and these are as follows:

a) Apparent amorphous content is not a (key) driver for performance and therefore does not need a control strategy to guarantee performance.
b) Data shows that apparent amorphous content is a driver for performance and therefore needs a control strategy.
c) The apparent amorphous content is typically below the detection limit and no indications are present for the amorphous content to be a key driver for the performance.

When data indicates that apparent amorphous content is a key driver for the performance (e.g. Fig. 3b), a control strategy for this parameter is required. Identification of the acceptable range in the apparent amorphous content during the manufacturing can be derived from the acceptable performance range. Important to note here is that the amorphous content typically reduces over time and that it is therefore desirable to work with maximum values for amorphous content only. Assurance of an acceptable amount of apparent amorphous content at the moment of use could be achieved for example by screening of samples before use, implementation of time restrictions between production and use, or exposure to specific conditions before use. Reporting of amorphous content by excipient suppliers could be supportive and justified as guidance, but does not absolve end users from establishing appropriate control strategy.

## 6 Regulatory landscape

Although the exact impact of amorphous lactose and/or the relevance on the efficiency of drug delivery to the lungs is unclear (Zeng et al., 2001), regulatory agencies acknowledge the potential relevance of amorphous content. Effort has also been made to draw the formulators attention towards amorphous content as a relevant parameter during DPI development (FDA, 2018).

The current monographs for lactose described in the European Pharmacopeia (EP), United States Pharmacopeia- National Formulary (USP-NF), Japanese Pharmacopeia (JP), Indian Pharmacopeia (IP) and the Chinese Pharmacopeia (CP) do not describe any mandatory testing for the apparent amorphous content (*Lactose Hydrate Monograph*, 2022; Lactose Monohydrate Monograph, 2021; Lactose Monograph, 2022; Lactose Monohydrate Monograph, 2020; Lactose Monohydrate Monograph, 2023). No reference to amorphous lactose can be found in any of these monographs, except for the USP-NF monograph on lactose monohydrate. This monograph includes only a note in the definition section to explain that lactose monohydrate “may contain varying proportions of amorphous lactose”.

Although the current regulatory landscape might not require testing of amorphous lactose, it is in scope for many discussions on future or revised versions. Examples on running discussions are those adopted by the USP-NF and CP (Moreton et al., 2020; Lactose for inhalation for CP2025, 2022), indicating the increased attention for the amorphous content of lactose, and will be discussed in more detail in the following sections. Although not specifically reviewed in this manuscript, it is reasonable to expect that other regulatory agencies are dealing with the same challenges and discussion points.

### 6.1 USP-NF monograph

USP-NF is continuously working with stakeholders to keep the content of their monographs and general chapters current. As part of the modernization program, it was acknowledged that the monograph for lactose monohydrate might not be fully aligned for inhalation applications. Although the original plan was to introduce a separate monograph for inhalation grade lactose, it was then decided that a separate monograph was not appropriate and that additional requirements for specific applications should be incorporated into the existing monographs. In 2020, the Excipients Monographs 2 Expert Committee of the USP-NF therefore proposed a revision of the monographs for anhydrous lactose and lactose monohydrate to cover the use of these ingredients in inhalation (and parenteral) dosage forms (Moreton et al., 2020). This stimuli article proposed that the revision should include additional quality testing and four performance tests for inhalation. The performance tests were related to non-standardized properties for the use in inhalation. Properties that are in scope of the discussion are the content of alpha and beta anomers, particle size distribution, particle shape and morphology and the amorphous content. No acceptance criteria are suggested for these parameters, as according to ‘General Notices 4.10. Monographs’ the link between excipient performance and medicinal product performance can only be determined by the excipient user (General Notices and Requirements, 2023), making it impossible for all possible limits of these tests to be included in a monograph.

The performance tests were proposed to be included in the ‘other requirements’ in the ‘labelling section’ in the monograph. This implies that only if a link between the excipient performance and medicinal product performance was established by the excipient user, the performance-related property should be determined. This approach is also in line with the control strategy recommended in the US FDA draft guidance for industry (2018) (FDA, 2018), whereby appropriate controls for excipients should be established only if a certain property of an excipient can affect the critical quality attribute of the final product.

In conclusion, a plan for adding amorphous content without any acceptance criteria to the monograph for inhalation applications was proposed, but it has not been finalized till this moment. Current status therefore remains that no testing for apparent amorphous content is described in the current editions of USP-NF monographs for lactose.

### 6.2 Chinese Pharmacopeia (CP) monograph

Chinese regulatory agencies also have running discussions with expert groups on lactose for inhalation use. The CP has proposed two separate monographs for inhalation grade lactose, namely ‘Lactose Monohydrate (for inhalation)’ and ‘Lactose (for inhalation)’ (Lactose Monohydrate Monograph, 2020). These proposed monographs for the CP 2025 do not list amorphous content as a parameter. Discussions are however ongoing on how to deal with amorphous lactose and if a parameter should be added to future versions.

The International Pharmaceutical Excipient Council (IPEC) Europe has suggested to the CP to include the amorphous content as part of a non-mandatory parameters section without acceptance criteria (Lactose for inhalation for CP2025, 2022). The amorphous content in that case should be considered relevant if studies as performed by the excipient user show the relevance of this parameter for the final performance of the formulation. In that case, excipient users and suppliers should agree on a practical and accurate test method and acceptance criteria. Without such a basis, the determination of such a parameter should however be omitted, as it would only add to the burden and to the cost for both excipient users and excipient suppliers.

### 6.3 Regulatory implications

When the apparent amorphous content is not identified as a critical parameter for the final product performance, it is recommended to exclude this parameter in a drug product registration. Inclusion of this parameter in that case would only add to the burden and to the cost for both excipient users and excipient suppliers. Exclusion of this parameters, on the other hand, might prevent unnecessary regulatory burden in the case of switching to a different excipient supplier. Where the supplier of an excipient was specified in an approved application, changing to a new supplier is done through the appropriate local regulatory procedure. If the excipient specification remains unchanged with respect to the critical material attributes as determined by the excipient user, the regulatory impact will be low (Kumar Nataraj et al., 2020). In the USA this would be mentioned in the annual report, and in Europe as a type 1 change. It is therefore critical to appropriately specify the excipient in the initial application and list specifications when required for final product quality. This criteria is in particular applicable to the amorphous content.

## 7 Conclusion

Amorphous lactose is a term used for all phases of lactose that do not have a defined crystalline structure. The chemical and physical stability of amorphous lactose is typically lower than that of crystalline lactose. The exact role of amorphous lactose fractions for the performance of a dry powder inhalation formulation however remains unclear. There are many complexities associated with the apparent amorphous content in lactose carriers used for DPIs. The apparent amorphous content in lactose is difficult to define, as the definition encompasses many different types of disorder. Different quantification methods are based upon a different property of amorphous lactose, which can result in different values. Additionally, the apparent amorphous content is susceptible to crystallization and therefore unstable over time. It can change during transport, storage and processing. Understanding the impact of amorphous content on the performance can only be obtained when the amorphous content is monitored during all processing steps. It is a challenging parameter that has the attention of the industry and an in-depth understanding and clear agreements between drug authorization holders and suppliers is critical.

## Funding details

Not applicable.

## CRediT authorship contribution statement

**Pauline H.M. Janssen:** Conceptualization, Investigation, Resources, Data curation, Writing – original draft, Writing – review & editing, Visualization, Supervision. **Lorina M.N. Bisharat:** Conceptualization, Investigation, Resources, Data curation, Writing – original draft, Writing – review & editing. **Marly Bastiaansen:** Conceptualization, Resources, Writing – review & editing.

## Declaration of Competing Interest

The authors report there are no competing interests to declare.

## Data Availability

Data will be made available on request.
